# Poly[[diaqua­hemi-μ_4_-oxalato-μ_2_-oxalato-praseodymium(III)] monohydrate]

**DOI:** 10.1107/S1600536809033947

**Published:** 2009-08-29

**Authors:** Ting-Hai Yang, Qiang Chen, Wei Zhuang, Zhe Wang, Bang-Yi Yue

**Affiliations:** a High-Tech Institute of Nangjing University, Changzhou 213164, People’s Republic of China

## Abstract

In the title complex, {[Pr(C_2_O_4_)_1.5_(H_2_O)_2_]·H_2_O}_*n*_, the Pr^III^ ion, which lies on a crystallographic inversion centre, is coordinated by seven O atoms from four oxalate ligands and two O atoms from two water ligands; further Pr—O coordination from tetra­dentate oxalate ligands forms a three-dimensional structure. The compound crystallized as a monohydrate, the water mol­ecule occupying space in small voids and being secured by O—H⋯O hydrogen bonding as an acceptor from ligand water H atoms and as a donor to oxalate O-acceptor sites.

## Related literature

For background to lanthanide oxalates and their preparation, see: Hansson (1970 ▶, 1972 ▶, 1973*a* ▶, 1973*b* ▶); Michaelides *et al.* (1988 ▶); Ollendorf & Weigel (1969 ▶); Steinfink & Brunton (1970 ▶); Trollet *et al.* (1998 ▶); Trombe (2003 ▶); Unaleroglu *et al.* (1997 ▶). For related structures, see: Trombe *et al.* (2004 ▶); Barrett Adams *et al.* (1998 ▶); Beagley *et al.* (1988 ▶).
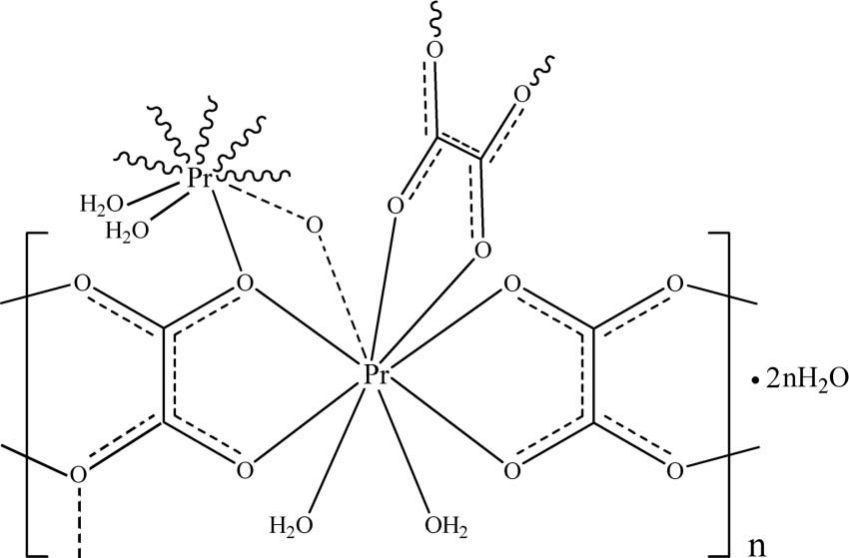


## Experimental

### 

#### Crystal data

[Pr(C_2_O_4_)_1.5_(H_2_O)_2_]·H_2_O
*M* *_r_* = 326.99
Triclinic, 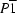

*a* = 6.0367 (12) Å
*b* = 7.6222 (15) Å
*c* = 8.9353 (18) Å
α = 98.330 (4)°
β = 99.814 (3)°
γ = 96.734 (4)°
*V* = 396.58 (14) Å^3^
*Z* = 2
Mo *K*α radiation
μ = 6.17 mm^−1^
*T* = 273 K
0.18 × 0.16 × 0.10 mm

#### Data collection

Bruker SMART APEX CCD diffractometer
Absorption correction: multi-scan (*SADABS*; Bruker, 2001 ▶) *T*_min_ = 0.341, *T*_max_ = 0.542
2140 measured reflections
1521 independent reflections
1450 reflections with *I* > 2σ(*I*)
*R*_int_ = 0.020

#### Refinement

*R*[*F* ^2^ > 2σ(*F* ^2^)] = 0.029
*wR*(*F* ^2^) = 0.075
*S* = 1.00
1521 reflections
118 parameters
H-atom parameters constrained
Δρ_max_ = 0.80 e Å^−3^
Δρ_min_ = −1.50 e Å^−3^

### 

Data collection: *SMART* (Bruker, 2007 ▶); cell refinement: *SAINT* (Bruker, 2007 ▶); data reduction: *SAINT*; program(s) used to solve structure: *SHELXS97* (Sheldrick, 2008 ▶); program(s) used to refine structure: *SHELXL97* (Sheldrick, 2008 ▶); molecular graphics: *DIAMOND* (Brandenburg, 1999 ▶); software used to prepare material for publication: *SHELXTL* (Sheldrick, 2008 ▶).

## Supplementary Material

Crystal structure: contains datablocks I, global. DOI: 10.1107/S1600536809033947/nk2001sup1.cif

Structure factors: contains datablocks I. DOI: 10.1107/S1600536809033947/nk2001Isup2.hkl

Additional supplementary materials:  crystallographic information; 3D view; checkCIF report

## Figures and Tables

**Table 1:** Hydrogen-bond geometry (Å, °)

*D*—H⋯*A*	*D*—H	H⋯*A*	*D*⋯*A*	*D*—H⋯*A*
O2*W*—H2*WB*⋯O1*W*	0.84	2.55	2.858 (7)	103
O1*W*—H1*WA*⋯O2^i^	0.84	1.96	2.694 (6)	146
O1*W*—H1*WA*⋯O3^ii^	0.84	2.60	3.235 (6)	133
O1*W*—H1*WB*⋯O3*W*^iii^	0.84	2.00	2.833 (6)	169
O2*W*—H2*WA*⋯O3*W*^iv^	0.84	1.98	2.807 (7)	166
O2*W*—H2*WB*⋯O3^v^	0.84	2.20	2.919 (6)	144
O3*W*—H3*WA*⋯O6^vi^	0.84	2.08	2.829 (7)	149
O3*W*—H3*WB*⋯O4^vii^	0.84	2.03	2.833 (6)	159

Symmetry codes: (i) 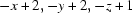; (ii) 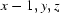; (iii) 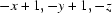; (iv) 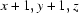; (v) 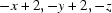; (vi) 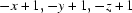; (vii) 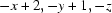.

**Fig. 1.**
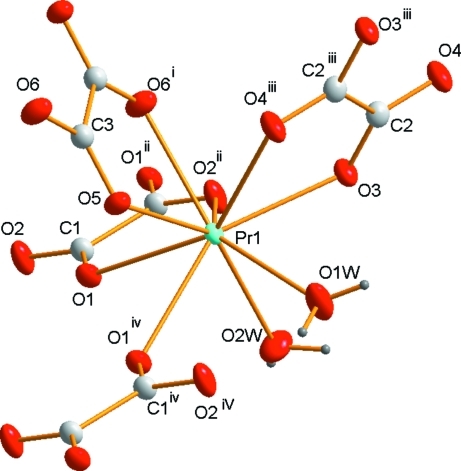
Molecular structure of (I), showing 50% probability displacement ellipsoids. [symmetry codes:(i) -x + 2,-y + 1,-z + 1; (ii) -x + 3,-y + 2,-z + 1; (iii) -x + 2,-y + 1,-z; (iv) -x + 2,-y + 2,-z + 1.

**Fig. 2.**
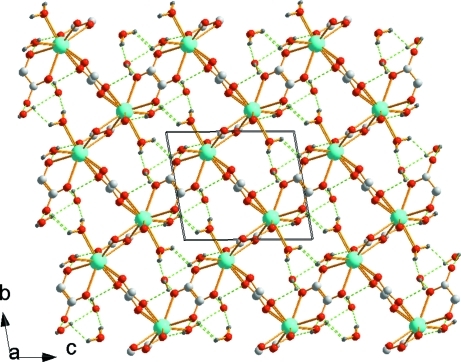
The unit cell packing diagram of (I).

## supplementary crystallographic information

### Comment

In the last decade considerable attention has been afforded to the structures and properties of lanthanide oxalates due to their ability to act as precursors of lanthanide oxides. Some single crystals of lanthanide oxalates, such as[Ln(C_2_O_4_)_3_(H_2_O)_4_].(H_2_O) (Ln = Sc or Yb), [Ln_2_(C_2_O_4_)_3_(H_2_O)_6_].4H_2_O (Ln = La, Ce, Pr or Nd) and [Nd_2_(C_2_O_4_)_3_(H_2_O)_6_] have been obtained either in silica gel (Ollendorff & Weigel, 1969), by hydrothermal reaction (Michaelides *et al.*, 1988) or by other methods (Hansson, 1970, 1972, 1973*a*,b; Unaleroglu *et al.*, 1997; Trollet *et al.*, 1998; Trombe, 2003). Crystals of [Ln(C_2_O_4_)(HC_2_O_4_)].4H_2_O (Ln = Er or Tm) were prepared by saturating a boiling solution of oxalic acid in 3 *M* H_2_SO_4_ with the lanthanide oxide and then slowly cooling to 273 K (Steinfink & Brunton, 1970). However, a few praseodymium oxalate complex has been reported. The present paper is concerned with a new crystal structure of praseodymium oxalate complex with a three-dimensional network structure.

The asymmetric unit of the title compound, [Pr(C_2_O_4_)_1.5_(H_2_O)_2_].(H_2_O), (I), is shown in Fig. 1. The Pr atom lies on an inversion centre and is coordinated by seven O atoms [O1, O1^iv^, O2^ii^, O3, O4^iii^, O5 and O6^i^] from four oxlate ligands, and two O atoms from two aqua ligands, thereby forming a slightly distorted PrO_9_ polyhedral coordination geometry. The Pr—O bond distances range from 2.451 (4) Å to 2.608 (3) Å, in agreement with those in compounds (Trombe *et al.* (2004); Barrett Adams *et al.* (1998)).

In the complex, the equivalent Pr atom are connected is coordinated by seven O atoms from four oxalate ligands and two O atoms from water ligands. Further Pr–O coordination from the tetradentate oxalate ligands forms a three-dimensional structure (Fig.2). The compound crystallized as a monohydrate; this water molecule occupies space in small voids and is secured by O–H···O hydrogen bonding as an acceptor from ligand water H-atoms and as a donor to oxalate O acceptor sites.

### Experimental

All solvents and chemicals were of analytical grade and were used without further purification. Pr(NO_3_)_3_^.^6H_2_O (0.05 mmol, 0.023 g), Na_2_C_2_O_4_(0.075 mmol, 0.011 g), and deionized water (10 ml) were mixed together. The mixture was sealed in a Teflon-line autoclave and then heated at 443 K for 5 d under autogenous pressure and then cooled to room temperature. Green crystals were obtained.

### Refinement

All non-hydrogen atoms were refined anisotropically. The water H atoms were located in a difference Fourier map and refined with a distance restraint of O-H = 0.83-0.85 Å, and with U_iso_(H) = 1.5U_iso_(O).

### Crystal data

**Table:**

[Pr(C_2_O_4_)_1.5_(H_2_O)_2_]·H_2_O	*Z* = 2
*M**_r_* = 326.99	*F*(000) = 310
Triclinic, *P*1	*D*_x_ = 2.738 Mg m^−^^3^
Hall symbol: -P 1	Mo *K*α radiation, λ = 0.71073 Å
*a* = 6.0367 (12) Å	Cell parameters from 905 reflections
*b* = 7.6222 (15) Å	θ = 3.3–28.3°
*c* = 8.9353 (18) Å	µ = 6.17 mm^−^^1^
α = 98.330 (4)°	*T* = 273 K
β = 99.814 (3)°	Block, green
γ = 96.734 (4)°	0.18 × 0.16 × 0.10 mm
*V* = 396.58 (14) Å^3^	

### Data collection

**Table:**

Bruker SMART APEX CCD diffractometer	1521 independent reflections
Radiation source: fine-focus sealed tube	1450 reflections with *I* > 2σ(*I*)
graphite	*R*_int_ = 0.020
φ and ω scans	θ_max_ = 26.0°, θ_min_ = 2.4°
Absorption correction: multi-scan (*SADABS*; Bruker, 2001)	*h* = −7→7
*T*_min_ = 0.341, *T*_max_ = 0.542	*k* = −9→8
2140 measured reflections	*l* = −10→11

### Refinement

**Table:**

Refinement on *F*^2^	Primary atom site location: structure-invariant direct methods
Least-squares matrix: full	Secondary atom site location: difference Fourier map
*R*[*F*^2^ > 2σ(*F*^2^)] = 0.029	Hydrogen site location: inferred from neighbouring sites
*wR*(*F*^2^) = 0.075	H-atom parameters constrained
*S* = 1.00	*w* = 1/[σ^2^(*F*_o_^2^) + (0.049*P*)^2^ + 1.09*P*] where *P* = (*F*_o_^2^ + 2*F*_c_^2^)/3
1521 reflections	(Δ/σ)_max_ = 0.001
118 parameters	Δρ_max_ = 0.80 e Å^−^^3^
0 restraints	Δρ_min_ = −1.49 e Å^−^^3^

### Special details

**Table:**

Geometry. All e.s.d.'s (except the e.s.d. in the dihedral angle between two l.s. planes) are estimated using the full covariance matrix. The cell e.s.d.'s are taken into account individually in the estimation of e.s.d.'s in distances, angles and torsion angles; correlations between e.s.d.'s in cell parameters are only used when they are defined by crystal symmetry. An approximate (isotropic) treatment of cell e.s.d.'s is used for estimating e.s.d.'s involving l.s. planes.
Refinement. Refinement of *F*^2^ against ALL reflections. The weighted *R*-factor *wR* and goodness of fit *S* are based on *F*^2^, conventional *R*-factors *R* are based on *F*, with *F* set to zero for negative *F*^2^. The threshold expression of *F*^2^ > σ(*F*^2^) is used only for calculating *R*-factors(gt) *etc*. and is not relevant to the choice of reflections for refinement. *R*-factors based on *F*^2^ are statistically about twice as large as those based on *F*, and *R*- factors based on ALL data will be even larger.

### Fractional atomic coordinates and isotropic or equivalent isotropic displacement parameters (Å^2^)

**Table:**

	*x*	*y*	*z*	*U*_iso_*/*U*_eq_	
Pr1	1.00780 (5)	0.80762 (4)	0.29489 (3)	0.01182 (13)	
C1	1.4452 (9)	1.0278 (7)	0.5703 (6)	0.0155 (11)	
C2	1.0808 (10)	0.5659 (7)	−0.0322 (7)	0.0163 (12)	
C3	0.8789 (9)	0.5155 (7)	0.5109 (6)	0.0157 (11)	
O1	1.2322 (6)	0.9964 (5)	0.5514 (4)	0.0156 (8)	
O2	1.5785 (7)	1.1009 (6)	0.6910 (5)	0.0220 (9)	
O3	1.1296 (7)	0.7232 (5)	0.0431 (5)	0.0195 (9)	
O4	1.1455 (7)	0.5077 (5)	−0.1515 (5)	0.0217 (9)	
O5	0.8004 (7)	0.6413 (5)	0.4552 (5)	0.0195 (9)	
O6	0.7818 (7)	0.4116 (6)	0.5849 (5)	0.0230 (9)	
O1W	0.6751 (7)	0.8292 (7)	0.0928 (5)	0.0310 (11)	
H1WA	0.5581	0.8534	0.1260	0.046*	
H1WB	0.6299	0.7771	0.0011	0.046*	
O2W	1.0612 (8)	1.0993 (6)	0.2071 (5)	0.0288 (10)	
H2WA	1.1653	1.1872	0.2227	0.043*	
H2WB	0.9752	1.1029	0.1239	0.043*	
O3W	0.4213 (9)	0.3694 (7)	0.2101 (6)	0.0420 (13)	
H3WA	0.4103	0.4339	0.2922	0.063*	
H3WB	0.5590	0.3823	0.2031	0.063*	

### Atomic displacement parameters (Å^2^)

**Table:**

	*U*^11^	*U*^22^	*U*^33^	*U*^12^	*U*^13^	*U*^23^
Pr1	0.01064 (19)	0.01179 (18)	0.01243 (18)	0.00024 (12)	0.00360 (12)	−0.00045 (12)
C1	0.012 (3)	0.016 (3)	0.019 (3)	0.001 (2)	0.005 (2)	0.001 (2)
C2	0.015 (3)	0.016 (3)	0.018 (3)	0.002 (2)	0.005 (2)	0.002 (2)
C3	0.016 (3)	0.016 (3)	0.014 (3)	0.002 (2)	0.004 (2)	0.000 (2)
O1	0.0075 (19)	0.019 (2)	0.020 (2)	0.0013 (15)	0.0047 (15)	−0.0003 (16)
O2	0.013 (2)	0.032 (2)	0.016 (2)	−0.0027 (17)	0.0036 (16)	−0.0062 (18)
O3	0.023 (2)	0.016 (2)	0.017 (2)	−0.0029 (16)	0.0072 (17)	−0.0033 (16)
O4	0.025 (2)	0.018 (2)	0.020 (2)	−0.0062 (17)	0.0118 (17)	−0.0028 (17)
O5	0.021 (2)	0.019 (2)	0.023 (2)	0.0084 (17)	0.0089 (17)	0.0085 (17)
O6	0.017 (2)	0.026 (2)	0.031 (2)	0.0070 (18)	0.0095 (18)	0.0138 (19)
O1W	0.015 (2)	0.053 (3)	0.023 (2)	0.010 (2)	0.0049 (18)	−0.005 (2)
O2W	0.029 (3)	0.021 (2)	0.035 (3)	−0.0021 (19)	0.001 (2)	0.013 (2)
O3W	0.029 (3)	0.050 (3)	0.038 (3)	−0.013 (2)	0.013 (2)	−0.017 (2)

### Geometric parameters (Å, °)

**Table:**

Pr1—O5	2.448 (4)	C2—C2^iii^	1.555 (11)
Pr1—O2W	2.466 (4)	C3—O5	1.248 (7)
Pr1—O6^i^	2.472 (4)	C3—O6	1.258 (7)
Pr1—O2^ii^	2.490 (4)	C3—C3^i^	1.548 (11)
Pr1—O1W	2.500 (4)	O1—Pr1^iv^	2.609 (4)
Pr1—O3	2.504 (4)	O2—Pr1^ii^	2.490 (4)
Pr1—O4^iii^	2.541 (4)	O4—Pr1^iii^	2.541 (4)
Pr1—O1	2.586 (4)	O6—Pr1^i^	2.472 (4)
Pr1—O1^iv^	2.609 (4)	O1W—H1WA	0.8413
C1—O2	1.243 (7)	O1W—H1WB	0.8417
C1—O1	1.258 (7)	O2W—H2WA	0.8412
C1—C1^ii^	1.546 (11)	O2W—H2WB	0.8372
C2—O4	1.238 (7)	O3W—H3WA	0.8386
C2—O3	1.263 (7)	O3W—H3WB	0.8400
			
O5—Pr1—O2W	142.99 (15)	O6^i^—Pr1—O1^iv^	120.77 (14)
O5—Pr1—O6^i^	65.89 (14)	O2^ii^—Pr1—O1^iv^	121.99 (13)
O2W—Pr1—O6^i^	142.54 (15)	O1W—Pr1—O1^iv^	77.27 (13)
O5—Pr1—O2^ii^	131.79 (14)	O3—Pr1—O1^iv^	146.78 (13)
O2W—Pr1—O2^ii^	71.54 (15)	O4^iii^—Pr1—O1^iv^	122.88 (13)
O6^i^—Pr1—O2^ii^	71.37 (14)	O1—Pr1—O1^iv^	65.40 (14)
O5—Pr1—O1W	97.65 (15)	O2—C1—O1	126.7 (5)
O2W—Pr1—O1W	70.26 (16)	O2—C1—C1^ii^	116.0 (6)
O6^i^—Pr1—O1W	142.27 (16)	O1—C1—C1^ii^	117.3 (6)
O2^ii^—Pr1—O1W	130.26 (15)	O4—C2—O3	126.8 (5)
O5—Pr1—O3	133.93 (13)	O4—C2—C2^iii^	117.5 (6)
O2W—Pr1—O3	78.07 (15)	O3—C2—C2^iii^	115.7 (6)
O6^i^—Pr1—O3	92.40 (14)	O5—C3—O6	126.5 (5)
O2^ii^—Pr1—O3	67.24 (13)	O5—C3—C3^i^	117.1 (6)
O1W—Pr1—O3	74.66 (14)	O6—C3—C3^i^	116.4 (6)
O5—Pr1—O4^iii^	70.30 (13)	C1—O1—Pr1	118.7 (3)
O2W—Pr1—O4^iii^	132.32 (15)	C1—O1—Pr1^iv^	123.3 (3)
O6^i^—Pr1—O4^iii^	69.96 (15)	Pr1—O1—Pr1^iv^	114.60 (14)
O2^ii^—Pr1—O4^iii^	114.58 (14)	C1—O2—Pr1^ii^	123.5 (4)
O1W—Pr1—O4^iii^	72.53 (15)	C2—O3—Pr1	121.8 (4)
O3—Pr1—O4^iii^	64.03 (13)	C2—O4—Pr1^iii^	120.5 (4)
O5—Pr1—O1	85.86 (13)	C3—O5—Pr1	120.4 (4)
O2W—Pr1—O1	81.79 (14)	C3—O6—Pr1^i^	119.7 (4)
O6^i^—Pr1—O1	77.20 (14)	Pr1—O1W—H1WA	115.2
O2^ii^—Pr1—O1	63.42 (12)	Pr1—O1W—H1WB	132.7
O1W—Pr1—O1	137.77 (14)	H1WA—O1W—H1WB	106.2
O3—Pr1—O1	130.36 (13)	Pr1—O2W—H2WA	136.4
O4^iii^—Pr1—O1	145.06 (13)	Pr1—O2W—H2WB	113.6
O5—Pr1—O1^iv^	67.14 (13)	H2WA—O2W—H2WB	107.3
O2W—Pr1—O1^iv^	76.00 (14)	H3WA—O3W—H3WB	107.4
			
O2—C1—O1—Pr1	−172.7 (5)	C2^iii^—C2—O3—Pr1	6.3 (8)
C1^ii^—C1—O1—Pr1	8.0 (8)	O5—Pr1—O3—C2	1.9 (5)
O2—C1—O1—Pr1^iv^	29.2 (8)	O2W—Pr1—O3—C2	−156.5 (4)
C1^ii^—C1—O1—Pr1^iv^	−150.1 (5)	O6^i^—Pr1—O3—C2	60.1 (4)
O5—Pr1—O1—C1	133.4 (4)	O2^ii^—Pr1—O3—C2	128.7 (5)
O2W—Pr1—O1—C1	−81.6 (4)	O1W—Pr1—O3—C2	−83.9 (4)
O6^i^—Pr1—O1—C1	67.2 (4)	O4^iii^—Pr1—O3—C2	−6.2 (4)
O2^ii^—Pr1—O1—C1	−8.2 (4)	O1—Pr1—O3—C2	135.3 (4)
O1W—Pr1—O1—C1	−129.8 (4)	O1^iv^—Pr1—O3—C2	−117.2 (4)
O3—Pr1—O1—C1	−15.0 (5)	O3—C2—O4—Pr1^iii^	−174.2 (5)
O4^iii^—Pr1—O1—C1	87.3 (4)	C2^iii^—C2—O4—Pr1^iii^	4.8 (9)
O1^iv^—Pr1—O1—C1	−159.9 (5)	O6—C3—O5—Pr1	−174.3 (5)
O5—Pr1—O1—Pr1^iv^	−66.72 (16)	C3^i^—C3—O5—Pr1	6.5 (8)
O2W—Pr1—O1—Pr1^iv^	78.30 (17)	O2W—Pr1—O5—C3	−154.9 (4)
O6^i^—Pr1—O1—Pr1^iv^	−132.92 (18)	O6^i^—Pr1—O5—C3	−6.5 (4)
O2^ii^—Pr1—O1—Pr1^iv^	151.7 (2)	O2^ii^—Pr1—O5—C3	−36.2 (5)
O1W—Pr1—O1—Pr1^iv^	30.1 (3)	O1W—Pr1—O5—C3	138.0 (4)
O3—Pr1—O1—Pr1^iv^	144.93 (15)	O3—Pr1—O5—C3	61.9 (5)
O4^iii^—Pr1—O1—Pr1^iv^	−112.8 (2)	O4^iii^—Pr1—O5—C3	69.7 (4)
O1^iv^—Pr1—O1—Pr1^iv^	0.0	O1—Pr1—O5—C3	−84.4 (4)
O1—C1—O2—Pr1^ii^	−171.6 (4)	O1^iv^—Pr1—O5—C3	−149.4 (4)
C1^ii^—C1—O2—Pr1^ii^	7.8 (9)	O5—C3—O6—Pr1^i^	−173.9 (4)
O4—C2—O3—Pr1	−174.7 (5)	C3^i^—C3—O6—Pr1^i^	5.3 (8)

Symmetry codes: (i) −*x*+2, −*y*+1, −*z*+1; (ii) −*x*+3, −*y*+2, −*z*+1; (iii) −*x*+2, −*y*+1, −*z*; (iv) −*x*+2, −*y*+2, −*z*+1.

### Hydrogen-bond geometry (Å, °)

**Table:**

*D*—H···*A*	*D*—H	H···*A*	*D*···*A*	*D*—H···*A*
O2W—H2WB···O1W	0.84	2.55	2.858 (7)	103
O1W—H1WA···O2^iv^	0.84	1.96	2.694 (6)	146
O1W—H1WA···O3^v^	0.84	2.60	3.235 (6)	133
O1W—H1WB···O3W^vi^	0.84	2.00	2.833 (6)	169
O2W—H2WA···O3W^vii^	0.84	1.98	2.807 (7)	166
O2W—H2WB···O3^viii^	0.84	2.20	2.919 (6)	144
O3W—H3WA···O6^ix^	0.84	2.08	2.829 (7)	149
O3W—H3WB···O4^iii^	0.84	2.03	2.833 (6)	159

Symmetry codes: (iv) −*x*+2, −*y*+2, −*z*+1; (v) *x*−1, *y*, *z*; (vi) −*x*+1, −*y*+1, −*z*; (vii) *x*+1, *y*+1, *z*; (viii) −*x*+2, −*y*+2, −*z*; (ix) −*x*+1, −*y*+1, −*z*+1; (iii) −*x*+2, −*y*+1, −*z*.

## References

[bb1] Barrett Adams, D. M. Y., Kahwa, I. A. & Mague, J. T. (1998). *New J. Chem* **22**, 919–921.

[bb2] Beagley, B., Pritchard, R. G., Evmiridis, N. P., Michailides, A. & Skoulika, S. (1988). *Acta Cryst.* C**44**, 174–175.

[bb3] Brandenburg, K. (1999). *DIAMOND* Crystal Impact GbR, Bonn, Germany.

[bb4] Bruker (2001). *SADABS* Bruker AXS Inc., Madison, Wisconsin, USA.

[bb5] Bruker (2007). *SMART* and *SAINT* Bruker AXS Inc., Madison, Wisconsin, USA.

[bb6] Hansson, E. (1970). *Acta Chem. Scand* **24**, 2969–2982.

[bb7] Hansson, E. (1972). *Acta Chem. Scand* **26**, 1337–1350.

[bb8] Hansson, E. (1973*a*). *Acta Chem. Scand* **27**, 823–834.

[bb9] Hansson, E. (1973*b*). *Acta Chem. Scand* **27**, 2852–2860.

[bb10] Michaelides, A., Skoulika, S. & Aubry, A. (1988). *Mater. Res. Bull.* **23**, 579–585.

[bb11] Ollendorf, W. & Weigel, F. (1969). *Inorg. Nucl. Chem. Lett* **5**, 263–269.

[bb12] Sheldrick, G. M. (2008). *Acta Cryst.* A**64**, 112–122. 10.1107/S0108767307043930 18156677

[bb13] Steinfink, H. & Brunton, G. D. (1970). *Inorg. Chem.* **9**, 2112–2117.

[bb14] Trollet, D., Rome, S. & Mosset, A. (1998). *Polyhedron*, **17**, 3977–3978.

[bb15] Trombe, J. C. (2003). *J. Chem. Crystallogr.* **33**, 19–26.

[bb16] Trombe, J. C. & Mohanu, A. (2004). *Solid State Sci.* **6**, 1403–1419.

[bb17] Unaleroglu, C., Zumreoglu-Karan, B. & Zencir, Y. (1997). *Polyhedron*, **16**, 2155–2161.

